# Childhood Exposure to Ambient Air Pollutants and the Onset of Asthma: An Administrative Cohort Study in Québec

**DOI:** 10.1289/ehp.1509838

**Published:** 2016-01-05

**Authors:** Louis-Francois Tétreault, Marieve Doucet, Philippe Gamache, Michel Fournier, Allan Brand, Tom Kosatsky, Audrey Smargiassi

**Affiliations:** 1 Department of Environmental and Occupational Health, School of Public Health University of Montreal, Montréal, Québec, Canada; 2 Direction de santé publique, Centre intégré universitaire de santé et de services sociaux (CIUSSS) Centre-Sud-de-l’Île-de-Montréal, Montréal, Québec, Canada; 3 Institut national de la santé publique du Québec, Québec, Québec, Canada; 4 Department of Medicine, Laval University, Québec, Québec, Canada; 5 British Columbia Centre for Disease Control, Vancouver, British Columbia, Canada; 6 Université de Montréal Public Health Research Institute, Montréal, Québec, Canada

## Abstract

**Background:**

Although it is well established that air pollutants can exacerbate asthma, the link with new asthma onset in children is less clear.

**Objective:**

We assessed the association between the onset of childhood asthma with both time of birth and time-varying exposures to outdoor air pollutants.

**Method:**

An open cohort of children born in the province of Québec, Canada, was created using linked medical–administrative databases. New cases of asthma were defined as one hospital discharge with a diagnosis of asthma or two physician claims for asthma within a 2 year period. Annual ozone (O3) levels were estimated at the child’s residence for all births 1999–2010, and nitrogen dioxide (NO2) levels during 1996–2006 were estimated for births on the Montreal Island. Satellite based concentrations of fine particles (PM2.5) were estimated at a 10 km × 10 km resolution and assigned to residential postal codes throughout the province (1996–2011). Hazard ratios (HRs) were assessed with Cox models for the exposure at the birth address and for the time-dependent exposure. We performed an indirect adjustment for secondhand smoke (SHS).

**Results:**

We followed 1,183,865 children (7,752,083 person-years), of whom 162,752 became asthmatic. After controlling for sex and material and social deprivation, HRs for an interquartile range increase in exposure at the birth address to NO2 (5.45 ppb), O3 (3.22 ppb), and PM2.5 (6.50 μg/m3) were 1.04 (95% CI: 1.02, 1.05), 1.11 (95% CI: 1.10, 1.12), and 1.31 (95% CI: 1.28, 1.33), respectively. Effects of O3 and PM2.5 estimated with time-varying Cox models were similar to those estimated using exposure at birth, whereas the effect of NO2 was slightly stronger (HR = 1.07; 95% CI: 1.05, 1.09).

**Conclusions:**

Asthma onset in children appears to be associated with residential exposure to PM2.5, O3 and NO2.

**Citation:**

Tétreault LF, Doucet M, Gamache P, Fournier M, Brand A, Kosatsky T, Smargiassi A. 2016. Childhood exposure to ambient air pollutants and the onset of asthma: an administrative cohort study in Québec. Environ Health Perspect 124:1276–1282; http://dx.doi.org/10.1289/ehp.1509838

## Introduction

Asthma is one of the most common childhood chronic diseases worldwide (Asher et al. 2006; Garner and Kohen 2008; Williams et al. 1999). Although the link between exposure to air pollution and exacerbations of asthma is well established (HEI 2010), the association is less clear between increased concentrations of air pollutants and asthma onset. Although some studies have found positive associations between long-term exposure to pollutants including nitrogen oxides (NO_2_), ozone (O_3_), and particulate matter with diameter ≤ 2.5 μm (PM_2.5_) and the onset of childhood asthma (Clark et al. 2010; Gehring et al. 2010; Jerrett et al. 2008), other studies have reported an inverse association (Oftedal et al. 2009) or no association (Gruzieva et al. 2013) with exposure previous to onset. Inconsistencies between the results of these studies may be attributable to chance and/or unrecognized selection and confounding bias. Inconsistencies may also arise from differences in methods used to identify incident cases or estimate exposure to air pollutants (Gruzieva et al. 2013), differences in susceptibility, in the mixture of pollutants, or exposure patterns among populations.

Uncertainties also remain with regard to the effect of air pollutants in early life on asthma onset in later childhood years (Clark et al. 2010; Gruzieva et al. 2013; McConnell et al. 2006). Children may be more susceptible than adults to developing asthma due to exposure to air pollution because of their higher prevalence of mouth breathing, their small respiratory tracts, their immature immune systems, their higher ventilation rate, or the larger proportion of the day spent outside (California Environmental Protection Agency and American Lung Association of California 2003; Gilliland et al. 1999).

The aim of this population-based birth cohort study was to determine the association between the onset of childhood asthma to exposure at birth as well as, on a time-varying basis, to selected outdoor air pollutants.

## Methods

### Description of the Cohort

We used the Québec Integrated Chronic Disease Surveillance System (QICDSS) to create an open birth cohort of children born in Québec between 1 April 1996 and 31 March 2011. We created an additional subcohort restricted to children born and living on the island of Montreal during the same period. The QICDSS is derived from the linkage of data from four administrative health databases (i.e., by health insurance number): Health Insurance Registry; medical services (services dispensed through physician claims in emergency rooms and in physician’s office); discharges from hospitals; and deaths. Information on deaths was obtained from the Québec registry of demographic events. The QICDSS contains information on health services for about 95% of the Québec population (Blais et al. 2014). The Health Insurance Registry contains demographic information for all children born and living in Québec and geographic information (birth date, sex, and address of residence according to the six-character Canadian postal code).

Our purpose was to use existing time-varying estimates of ambient concentrations of O_3_, NO_2_, and PM_2.5_ and link these estimates to the residences of children during the follow-up period. Addresses are updated on the Health Insurance Registry as follows: When children move, parents or guardians are supposed to submit their new address to the Registry, but this is done on a voluntary basis. Additionally, a mandatory address update is required every 4 years to renew an individual’s health insurance card.

Air pollution levels for each year were assigned to the centroid of the six-character postal code where each child lived. Annual average NO_2_ levels were available for the Montreal Island subcohort only (from 1996 to 2006), whereas values for O_3_ and PM_2.5_ were assigned to postal codes throughout the province. For O_3_, annual average concentrations for each postal code (based on the average value measured in June–August of each year) were assigned for 1999–2011. A constant value for PM_2.5_ concentration was assigned to each postal code based on the estimated mean value during 2001–2006, which was assumed to apply throughout the study period (1996–2011). Children who moved to a different postal code were assigned pollutant exposures consistent with the new postal code from the day their address change took effect. Thus levels of air pollutants at a postal code centroid were applied to all residences in the postal code. When a child moved to a different postal code, the child’s exposure was changed to reflect the new postal code from the day the address change took effect. In urban areas, six-character postal codes often correspond to a single segment of road in which < 50 individuals live (with an average of 10,038 m^2^ and 349,910 m^2^ in the Montreal region and Quebec province respectively); in rural areas, these postal codes can cover a large territory. Centroids for postal codes were taken from the Canada Postal Code Conversion Files (Statistics Canada 2002, 2006, 2011).

### New Cases of Asthma

New cases of asthma were identified from either any hospital discharge showing a diagnosis of asthma (in any diagnostic field) or two physician claims for asthma (visits to the emergency room or physician’s office) occurring within a 2-year period (indexing occurred on the second visit). Gershon et al. (2010) reported that the sensitivity of this definition in Canadian children was 89% and its specificity 72%. This definition is currently used in Québec as well as Canada by public health institutions for asthma surveillance.

### Estimation of Air Pollutant Levels

We used annual (from 1996 through 2006) mean NO_2_ levels (parts per billion) estimated on a 5 m × 5 m grid for the island of Montreal (the subcohort of the Québec cohort) with a land use regression model developed by Crouse et al. (2009). Briefly, NO_2_ levels were measured in 133 locations on the island of Montreal with passive diffusion samplers during three seasons in 2005 and 2006. As reported by Crouse et al. (2009), NO_2_ levels for this period ranged from 2.6 ppb to 31.5 ppb (Crouse et al. 2009). NO_2_ levels before 2005 were derived from a back-extrapolation of the model. The extrapolation method involved multiplying the modeled NO_2_ levels by the site-specific ratio of past concentrations from fixed-site monitors of the Canadian National Air Pollution Surveillance (NAPS) network (Chen et al. 2010).

We estimated average summer (June–August) O_3_ levels (in ppb) at the centroid of each postal code using a Bayesian maximum entropy model described by Adam-Poupart et al. (2014) for the years 1999 through 2011, which were available for the study. O_3_ levels were estimated using a geographical interpolation from a combination of measured levels at the NAPS stations in Québec and estimates from a land-use mixed-effects model. The land-use mixed-effects model, with a 1 km × 1 km grid, was developed with the NAPS data and road network, meteorological data, and latitude. Ozone levels were estimated only for postal codes where land-use mixed-effects model estimates were available (23% of the children had no estimated exposure to O_3_ at their home address during their follow-up). We used summer O_3_ levels (parts per billion) as proxy for yearly exposure to O_3_ levels.

Mean PM_2.5_ levels for each postal code during 2001–2006 were derived using satellite imagery (van Donkelaar et al. 2010) and were assumed to be constant throughout the study period (1996–2011). van Donkelaar et al. (2010) calculated column aerosol optical depth (AOD) measurements using satellite instruments (Multiangle Imaging Spectroradiometer and Moderate Resolution Imaging Spectroradiometer). They then converted AOD measurements into surface PM_2.5_ levels using the global chemical transport model GEOS-Chem (van Donkelaar et al. 2010). Concentrations of PM_2.5_ (micrograms per cubic meter) were estimated for a grid of 10 km × 10 km. PM_2.5_ concentrations were then assigned to each postal code based on the grid in which the centroids of the postal codes were found.

### Socioeconomic Status

Because socioeconomic status of subjects was not available on an individual basis, we assessed its effect using an area-wide variable that represents “deprivation” (Pampalon et al. 2009). This index, based on six indicators taken from the Canadian census, is grouped into two components to assess material and social deprivation. The Pampalon index covers > 97% of all residential areas of Québec at the smallest census division (dissemination area level). Dissemination areas sometimes encompass multiple postal codes in the urban regions of our study area. Pampalon deprivation index values for 1996–1998 were assigned based on the 1996 census, values for 1999–2003 were based on the 2001 census, and values for 2004–2011 were based on the 2006 census.

### Statistical Analysis

We used Cox proportional hazard models to estimate hazard ratios (HRs) for exposures to PM_2.5_ (from 1996 through 2011), O_3_ (from 1999 through 2011), and NO_2_ (from 1996 through 2006, for the island of Montréal) at the residence of the children ascribed to their year of birth. Time-varying exposure models were also developed using time-varying annual average exposure level. We performed our analyses with age as our time axis (i.e. from birth to the end of follow-up), as suggested by Thiébaut and Bénichou (2004), and we used days as the unit of analysis. For the time-varying models, annual exposures assigned to each child varied depending on the year (for NO_2_ and O_3_), and, for children who moved during the study period, according to the annual NO_2_, O_3_, or PM_2.5_ exposure assigned to each postal code where the child resided during the study period, according to the date on which their address change took effect. Throughout our analyses, individuals were censored when they moved out of the province, died, or reached the age of 13 years. We express HRs per interquartile range (IQR), based on the total exposure distribution for each pollutant over the entire study period and increase in exposure levels and report 95% confidence intervals (CIs). Furthermore, ties in failure time were handled by the Efron method.

The linearity of the relation with continuous exposure variables was assessed using restricted cubic splines with three knots, with knots positioned according to the recommendations of Harrell (2001) (quantiles 0.10, 0.50, and 0.90), and the statistical significance of the nonlinear term was assessed with likelihood ratio tests. The proportional hazard assumption was assessed through the examination of the weighted and scaled Schoenfeld residuals in order to assess evident trend with time.

For each pollutant we estimated *a*) crude associations, associations adjusted for *b*) sex and quintiles of the Pampalon deprivation index, and associations adjusted for *c*) sex, quintiles of the Pampalon index, and year of birth in the cohort.

We performed an indirect adjustment for secondhand smoke (SHS) for the Montreal subcohort by using a strategy proposed by Steenland and Greenland (2004) and adapted by Villeneuve et al. (2011) for continuous exposures. To perform this analysis, we used area-specific (i.e., postal code) prevalence of at-home childhood exposure to SHS as was taken from a 2006 survey conducted in Montreal (Değer et al. 2010), as well as a rate ratio representing the association between childhood asthma and SHS from a meta-analysis (*n* = 20) (Tinuoye et al. 2013). The methods used for this analysis are described in detail in Supplemental Material, “Indirect adjustment for second hand smoke.”

We also performed the following sensitivity analyses: *a*) excluding regions (North of Quebec, Outaouais, the North Shore, the Gaspésie, and the Magdalene) where, based on current practices, health services may be underreported (because physicians in those regions are not paid by medical act or because residents are likely to use the health care system of another province); *b*) restricting the analysis to subjects who did not move during follow-up; and *c*) using an alternative definition of asthma onset. For this definition, cases occurring before the age of 5 years had to be confirmed with a hospitalization or a physician claim occurring at the age of 5 years or later for a child to be considered as asthmatic. This analysis was restricted to children who were born before 2002 to ensure they could be followed to their tenth birthday. To assess whether air pollutants are implicated in the well-known disparities in the incidence of asthma between boys and girls, and between rural and urban regions (i.e., regions with population numbers > 100,000 inhabitants were considered urban) (Garner and Kohen 2008; Nicolai et al. 2003), we performed regressions stratified by sex or region. The effect modification between strata was assessed using a Wald homogeneity test.

All analyses were performed with SAS 9.2 (SAS Institute Inc.) except for the indirect adjustment for tobacco exposure that was performed with R (version 3.1.0 with packages mvtnorm and mcsm) (R Core Team 2013). The project was carried out in the context of the Quebec ministerial health surveillance plan. The use of the QICDSS has been approved by the government for agencies managing databases, the Research Ethics Board of public health and the “Commission d’accès à l’information (CAI).”

## Results

**Figure 1:**
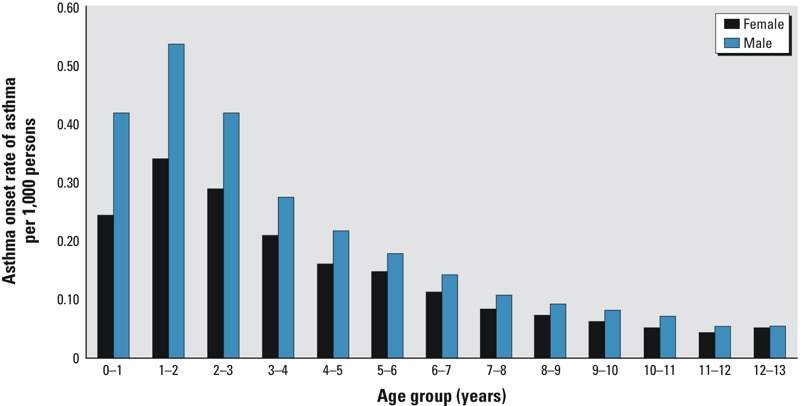
Asthma onset rate of asthma by sex and age group in the Québec cohort for the years 1996 to 2011.

Between 1996 and 2011, 1,183,865 children born in the province of Québec were followed for a total of 7,752,083 person-years. Of those, 2,355 children were censored during follow-up because of death and 64,531 moved out of the province. The majority of follow-up occurred before the age of 6 years (60.16%; Table 1). For the Montreal subcohort, 319,356 children were followed, contributing 1,651,294 person-years of follow-up. The distribution of the follow-up varied slightly from the rest of the province. A greater proportion of follow-up time accrued to younger children in the Montreal subcohort than in the entire Québec cohort. The Montreal subcohort showed greater disparity of social and material deprivation compared with the Québec cohort. During the follow-up period, 162,752 children were diagnosed with asthma in the provincial cohort, with asthma onset occurring more frequently at younger ages (Figure 1), and most commonly identified using physician claims (data not shown). As shown in Figure 1, asthma onset rates peaked in the 1- to 2-year-old age group, in the provincial cohort, for both males and females, and then diminished with age. Furthermore, the asthma onset rate was consistently higher in male versus females; however, this difference was attenuated with age.

**Table 1:** Descriptive statistics of study participants, for the provincial cohort and the Montreal Island subcohort, 1996–2011.

Characteristics	Québec (*n* = 1,183,865)	Montreal (*n* = 319,356)
Follow-up (person-years)	7,752,083	1,651,294
Number of person-years per age-group (%)
< 1	1,172,952 (15.13)	302,357 (18.31)
1–5	3,491,058 (45.03)	788,328 (47.74)
6–12	3,088,072 (39.84)	560,610 (33.95)
Male (%)	51.2	51.2
Pampalon material deprivation index (%)
1 (least deprived)	19.70	23.26
2	20.17	17.02
3	19.68	16.65
4	19.78	18.51
5 (most deprived)	20.64	24.56
Pampalon social deprivation index (%)
1 (least deprived)	19.55	13.63
2	20.20	13.31
3	19.73	16.32
4	20.29	27.03
5 (most deprived)	20.20	29.70

Distributions of NO_2_, O_3_, and PM_2.5_ between the birth address and the time-varying exposure during the follow-up were similar (Table 2). Concentrations of NO_2_ were available for the years 1996–2006 in the Montreal subcohort, whereas O_3_ and PM_2.5_ levels were available for the whole province, respectively, for 1999–2010 and 1996–2011. The time-varying means were slightly lower than the mean at the birth address. Average levels of PM_2.5_ at the birth address were higher in the Montreal subcohort (13.65 μg/m^3^) than in the Québec cohort (9.86 μg/m^3^), whereas O_3_ average levels were only slightly lower in the Montreal subcohort [31.55 ppb vs. 32.07 ppb for Montreal subcohort and Québec, respectively (see Table S1)]. Furthermore, the range of exposure is much more restricted for PM_2.5_ for the Montreal subcohort than for the Quebec cohort. The smaller range of exposure in the Montreal subcohort could be explained by the limited number of PM_2.5_ exposure grids on the Montral Island.

**Table 2:** Distributions of estimated levels of NO_2_, PM_2.5_, and O_3_ at both the time-varying and the birth address.*^a^*

Pollutant level	Birth address			Time-varying exposures		
	NO_2_^*b*^	O_3_^*c*^	PM_2.5_^*d*^	NO_2_^*b*^	O_3_^*c*^	PM_2.5_^*d*^
Minimum	4.47	12.19	2.32	4.47	12.19	2.32
25%	12.67	30.54	6.93	12.02	30.42	6.85
50%	15.07	32.19	9.51	14.35	32.04	9.24
75%	18.12	33.76	13.42	17.29	33.68	13.34
Maximum	35.90	43.12	14.85	39.36	43.39	14.85
Interquartile range	5.45	3.22	6.50	5.27	3.26	6.53
Mean	15.51	32.07	9.86	14.80	31.97	9.58
^***a***^Exposures based on annual levels. ^***b***^Restricted to the Montreal subcohort and for the years 1996–2006 (ppb). ^***c***^For the years 1999–2010 (ppb). ^***d***^For the years 1996–2011 (μg/m^3^).						

Table 3 presents crude and adjusted HRs per IQR increase of NO_2_ (5.45 ppb, *n* = 216,746), O_3_ (3.22 ppb, *n* = 829,277), and PM_2.5_ (6.50 μg/m^3^, *n* = 1,133,938) levels at the home address at birth. Because the nonlinear models did not present a statistically significant better fit than the linear models, all associations were considered linear (data not shown). Controlling for sex and deprivation indices decreased the crude associations observed for the Montreal subcohort for NO_2_ from 1.07 (95% CI: 1.06, 1.08) to 1.04 (95% CI: 1.02, 1.05), but did not alter the relations with O_3_ or PM_2.5_. Controlling for the year of birth in addition to the covariates of previous models decreased the association with O_3_ in the Québec cohort (HR from 1.11; 95% CI: 1.10, 1.12 to 1.06; 95% CI: 1.05, 1.07) and removed the association with NO_2_ in the Montreal subcohort (HR = 0.99; 95% CI: 0.97, 1.01).

**Table 3:** Associations between asthma onset and air pollutant levels at the birth address, per inter­quartile range increase of pollutant levels.*^a^*

Pollutant	Sample size	Interquartile range	Hazard ratios (95% CI)		
			Crude	Model 1^*b*^	Model 2^*c*^
NO_2_^*d*^	216,746	5.45 ppb	1.07 (1.06–1.08)*	1.04 (1.02–1.05)*	0.99 (0.97–1.01)
O_3_^*e*^	829,277	3.22 ppb	1.10 (1.09–1.11)*	1.11 (1.10–1.12)*	1.06 (1.05–1.07)*
PM_2.5_^*f*^	1,133,938	6.50 μg/m^3^	1.29 (1.27–1.31)*	1.31 (1.28–1.33)*	1.32 (1.30–1.33)*
^***a***^Exposures based on annual levels. ^***b***^Associations adjusted for sex and indexes of social and material deprivation. ^***c***^Associations adjusted for year of birth, sex, and indexes of social and material deprivation. ^***d***^Restricted to the Montreal subcohort, for the years 1996–2006. ^***e***^For the years 1999–2010. ^*f*^For the years 1996–2011. *p < 0.001.					

Adjustments for exposure to SHS in the Montreal subcohort, of associations between air pollutant levels at the residence of birth and asthma onset in children (Table 4) yielded similar HR estimates to the ones presented in Table 3, although with larger confidence intervals. For example, for the association for NO_2_ in the Montreal subcohort, the HR with indirect adjustment for SHS was 1.04 (95% CI: 1.01, 1.06 *n* = 216,746), whereas the HR without the adjustment was 1.04 (95% CI: 1.02, 1.05). The respective distribution of the bias factor can be observed in Figure S1. These associations in Table 4 are presented for an IQR based on the Montreal subcohort exposure distribution.

**Table 4:** Indirect adjustment for exposure to secondhand smoke, of associations between air pollutant levels at the birth address and asthma onset in children of the Montreal Island, per interquartile range increase in air pollutant levels.*^a^*

Pollutant	Sample size	Interquartile range	Hazard ratios (95% CI)^*b*^	
			Model 1	Indirect adjustment for secondhand smoke
NO_2_^*c*^	216,746	5.45 ppb	1.04 (1.02–1.05)*	1.04 (1.01–1.06)
O_3_^*d*^	177,187	3.09 ppb	1.11 (1.10–1.13)*	1.11 (1.09–1.13)*
PM_2.5_^*c*^	218,298	0.99 μg/m^3^	1.04 (1.03–1.05)*	1.04 (1.02–1.06)*
^***a***^Exposures based on annual levels for the Montreal subcohort. ^***b***^Associations adjusted for sex and indexes of social and material deprivation. ^***c***^For the years 1996–2006. ^***d***^For the years 1999–2006. *p < 0.001.				

Table 5 presents crude and adjusted HRs per IQR increase for NO_2_ (5.27 ppb), O_3_ (3.26 ppb), and PM_2.5_ (6.53 μg/m^3^) based on time-varying exposure during the follow-up period. Here too, nonlinear models did not have a statistically significant better fit than the linear models (data not shown), thus all associations were considered linear. Controlling for deprivation indices decreased the crude associations observed for the Montreal subcohort for NO_2_ from 1.10 (95% CI: 1.08, 1.12) to 1.07 (95% CI: 1.05, 1.09), but did not alter the relations with the two other air pollutants. Controlling for the year of birth decreased the association with O_3_ and NO_2_ (to 1.07; 95% CI: 1.06, 1.08 and 1.04; 95% CI: 1.03, 1.06, respectively). Associations obtained for the time-varying residential air pollutant levels (Table 5), did not differ greatly from those performed with pollutant levels at the home address at birth (Table 3).

**Table 5:** Associations between asthma onset and time-varying air pollutant levels, per interquartile range increase in pollutant levels at the residential address.*^a^*

Pollutant	Sample size	Interquartile range	Hazard ratios (95% CI)		
			Crude	Model 1^*b*^	Model 2^*c*^
NO_2_^*d*^	216,746	5.27 ppb	1.10 (1.08–1.12)*	1.07 (1.05–1.09)*	1.04 (1.03–1.06)*
O_3_^*e*^	829,277	3.26 ppb	1.10 (1.09–1.11)*	1.13 (1.11–1.14)*	1.07 (1.06–1.08)*
PM_2.5_^*f*^	1,133,938	6.53 μg/m^3^	1.31 (1.30–1.33)*	1.32 (1.31–1.33)*	1.33 (1.31–1.34)*
^***a***^Exposures based on annual levels. ^***b***^Associations adjusted for sex and indexes of social and material deprivation. ^***c***^Associations adjusted for year of birth, sex, indices of social and material deprivation. ^***d***^Restricted to the Montreal subcohort, 1996 to 2006. ^***e***^For the years 1999–2010. ^***f***^For the years 1996–2011. *p < 0.001.					

The exclusion of regions of the province where the use of health services may be underreported provided similar results to the ones presented in Tables 3 and 5 for the exposure at birth (see also Table S2). Results also remained unchanged when analyses were restricted to individuals in the provincial cohort who did not move (43% of subjects) during the follow-up for O_3_ and PM_2.5_ (see Table S3)_._ However, the CI for the association with NO_2_ among nonmovers in the Montreal subcohort was larger because only 26% did not move during follow-up (HR for time-varying NO_2_ exposure increase of 5.27 ppb = 1.04; 95% CI: 1.01, 1.06). In sensitivity analyses performed with asthma onsets confirmed at ages 5 years or later, 49.6% of the children diagnosed before 5 years in the original cohort were reconfirmed as asthmatic (see Table S4). When compared with HRs for the entire cohort adjusted for sex and deprivation, HRs for reconfirmed asthmatics were lower for PM_2.5_, higher for O_3_, and nearly identical for NO_2_ compared with those presented in Tables 3 and 5. HRs for PM_2.5_, NO_2_, and O_3_ at the residence of birth were 1.24 (95% CI: 1.22, 1.26) 1.06 (95% CI: 1.03, 1.14), and 1.21 (95% CI: 1.19, 1.22), respectively. Associations were similar between boys and girls for all pollutants and for time-varying exposures and exposures at birth, with Wald homogeneity test *p*-values > 0.27 (see Table S5). HRs for children in rural regions were slightly higher than corresponding estimates for children in urban areas, though differences were not statistically significant (Wald homogeneity test *p*-values > 0.18) (see Table S6).

## Discussion

Our study showed significant associations between children’s asthma onset and PM_2.5_, NO_2_, and O_3_ residential levels both for levels estimated at children’s birth addresses and for time-varying residential levels in Québec. Associations for an IQR increase, adjusted for sex and social and material deprivation, were slightly stronger for PM_2.5_ and O_3_ than for NO_2_. In this study the more widely distributed secondary pollutants (i.e., PM_2.5_ and O_3_) have a stronger association with asthma onset in children than primarily traffic-related emissions such as NO_2_.

We present two confounder models in the results. The first was adjusted for sex and deprivation, whereas the second was adjusted for the same variables as well as the year of birth. Adjusting for the year of birth did not cause important alteration of the HRs for PM_2.5_ and O_3_, but the association with exposure to NO_2_ in the first year of life disappeared. This could be explained by the fact that a backward extrapolation was used to estimate NO_2_ concentrations before 2005. Because a decrease of NO_2_ is observed through time, year of birth would be a predictor of the NO_2_ exposure at birth and not a confounder. We cannot, however, deny the possibility that year of birth is a proxy for an important confounder and that the association presented in model 1 is spurious.

One interpretation of our finding that air pollution is associated with the onset of asthma is that long-term air pollutant exposure in early life induces asthmatic tendencies in children who otherwise would not have been asthmatic. Another interpretation is that associations are attributable to the exacerbation of mild or subclinical asthma in children to the point where medical services are used (Anderson et al. 2013). The design of this study precludes us from finding the mechanism underlying these findings. However, we can conclude that long-term exposures were associated with increased use of medical services for asthma in children, which is in itself an outcome of interest for public health.

Many studies published to date have assessed the association between exposure to air pollutants at the birth address and asthma onset in children. Our point estimates are in line with those of most birth cohorts that assessed associations with air pollutant levels in the first year of life. Gruzieva et al. (2013) reported odds ratios of 1.34 (95% CI: 0.80, 2.23) per 7.2 μg/m^3^ of PM_10_ (particulate matter with median diameter ≤ 10 μm), and 1.21 (95% CI: 0.79, 1.84) per 46.8 μg/m^3^ of NO_x_ (nitrogen oxides) over a 12-year follow-up. In the PIAMA (Prevention and Incidence of Asthma and Mite Allergy) cohort with an 8-year follow-up, odds ratios for associations between asthma onset and birth address levels of PM_2.5_ and NO_2_ were 1.28 (95% CI: 1.10, 1.49) per 3.2 μg/m^3^ and 1.19 (95% CI: 1.05, 1.34) per 10.4 μg/m^3^, respectively (Gehring et al. 2010). Other publications from the PIAMA cohort with shorter follow-up time reported similar point estimates, although most were not statistically significant (Brauer et al. 2002, 2007). Krämer et al. (2009) also reported a positive association between exposure to NO_2_ at the birth address and asthma onset in children (HR = 1.17; 95% CI: 0.86, 1.56, per 9 μg/m^3^). A Canadian study (Clark et al. 2010), reported associations between PM_2.5_ and NO_2_ exposure in the first year of life, calculated with a land use regression analysis, and asthma onset of 1.01 (95% CI: 0.99, 1.03) per 1 μg/m^3^ and 1.13 (95% CI: 1.04, 1.23) per 10 μg/m^3^, respectively. In contrast, one study performed by Oftedal et al. (2009) found a negative effect of exposure to NO_2_ in the first year of life on the risk of childhood asthma onset before the age of 4 years. However this study was notably different than ours on some key aspects. First, Oftedal et al. (2009) used parent-reported asthma whereas we used medical records. Second, this negative association was found only for children < 4 years of age and not in older age groups.

Our results are also in agreement with those studies that assessed time-varying exposure to air pollutants, beside exposure at birth. Using the Southern California Children’s Health Study, Jerrett et al. (2008), McConnell et al. (2010), and Shankardass et al. (2009) reported HRs of 1.29 (95% CI: 1.07, 1.56) per 6.2-ppb increase of measured NO_2_, 1.48 (95% CI: 1.19, 1.85) per 17.4-μg/m^3^ increase of PM_2.5_ and 1.31 (95% CI: 1.07, 1.61) per 21-ppb increase of traffic-related pollutants. In Boston, Clougherty et al. (2007) presented an association with annual values of NO_2_ 1.17 (95% CI: 0.94, 1.46) per 4.3-ppb increase in the year of diagnosis. Two studies (Gruzieva et al. 2013; Oftedal et al. 2009) looked at the effect of exposure to pollutants at birth and annually. As with our study, in both cases annual associations were similar to those with exposure in the first year of life, even though Oftedal et al. (2009) found a protective effect. This may be related to the fact that time-varying air pollutant levels and pollutant levels at the birth address are highly correlated.

Our results and most of those of previous cohort studies are concordant, even if they differ on a number of methodological aspects. First, most studies above used questionnaires or interviews [except Clark et al. (2010)] to identify new cases of asthma, whereas we used medical–administrative health data. In consequence, these studies did not have a precise time of onset. Other studies (Brauer et al. 2002, 2007; Clark et al. 2010; Clougherty et al. 2007; Gehring et al. 2010; Gruzieva et al. 2013) used logistic regressions or generalized estimation equations, whereas some studies (Jerrett et al. 2008; Krämer et al. 2009; McConnell et al. 2010; Shankardass et al. 2009) used Cox models. As opposed to ours, none of the previously noted studies were able to use the precise day of asthma diagnosis in their analyses. Nonetheless, the use of questionnaire gave the possibility to researchers to adjust for covariates at the individual level, such as family history of asthma and exposure to SHS. Such information was not available in our database. Finally a wide array of methodology has been used to estimate air pollution exposure. A study by Jerrett et al. (2008) used measures of exposure from monitors placed outside the home of the children whereas other studies modeled exposure with land use regression or dispersion models.

Our study has numerous strengths. First, the cohort used is based on linked medical–administrative databases that cover nearly the entire population of Québec; because the population of Québec has universal access to health care, this virtually eliminates the likelihood of selection bias. Second, the large population and the length of the follow-up provide sufficient statistical power to detect small effects. Third, exposure to NO_2_ and O_3_ was assigned at the residential postal code with spatially explicit models of pollution dispersion, allowing us to take the geographical spread of air pollutants into account at a relatively small scale in urban regions. Finally, we were able to ascertain the residential history of the children and to assess the temporal variation of exposures to NO_2_ and O_3_ in order to assess modifications of exposure during the follow-up period.

Still, there are several limitations in this study. First, individual exposure was modeled and not measured through the follow-up, so the quality of the associations depends on the quality of the exposure models. All associations reported in this study were estimated according to the exposure at the centroid of the residential postal code. This assumes that children would stay at home all day. Because a large proportion of a child’s day can be spent outside the home (e.g., at school), where exposure to air pollutants might differ, misclassification bias may have been introduced in our study. Additionally, summer average O_3_ levels were used to estimate annual averages. Because summer O_3_ levels are higher than winter levels (Environment Canada 1999) in Canada, we may have overestimated annual average levels. Furthermore, although postal codes circumscribe a relatively small area in urban regions, postal codes may include much larger areas in rural regions. This difference in postal code size could lead to a degree of higher imprecision in exposure estimation in regions of the province that are less densely populated. Another source of exposure imprecision is the resolution at which models estimated air pollutant levels. Although levels of NO_2_ and O_3_ were estimated at a relatively small scale, PM_2.5_ was estimated at a larger scale (10 km × 10 km), encompassing several postal codes especially in urban areas. Thus we had access only to information on the urban variability of NO_2_ and O_3_ levels. Even though smaller-scale estimates of PM_2.5_ levels would have been preferable, this secondary pollutant is known to have a more homogenous local distribution than NO_2_ levels (Monn 2001). Furthermore, PM_2.5_ exposure for each year at each postal code during the follow-up (from 1996 to 2011) was assigned the average concentration from 2001 through 2006, which prevented us from accounting for temporal variation in PM_2.5_ exposure, except for variation that occurred when children moved from one postal code to another during follow-up. However, for the North American east coast (south of the province of Québec), only a small decrease in trends of satellite-derived annual PM_2.5_ was observed for the 1998–2012 period (Boys et al. 2014). Finally, we estimated exposure with imprecision in our models; measurement errors are expected predominantly of Berkson type, which produces little to no bias in point estimates (Baker et al. 1999).

A second limitation is that we identified incident cases of asthma based on medical–administrative data and thus possibly introduced information bias. Despite the lack of published consensus on the identification of cases of asthma with Canadian administrative databases, we applied a definition commonly used for surveillance purposes (Gershon et al. 2010) that has been validated in adults (Gershon et al. 2009). The age- and sex-specific asthma onset rates presented in Figure 1 are similar to those reported by the Ontario Asthma Surveillance Information System Database (Gershon et al. 2010). Nonetheless, the diagnosis of asthma in children < 5 years of age is challenging because lung function measurements are not reliable. Asthma diagnoses for those children are often based only on family history and symptom patterns (Pedersen et al. 2011). Thus, early transient wheeze from viral infection could be misdiagnosed as asthma onset. Although a diagnosis of asthma may be less reliable for this age group, our sensitivity analyses show that associations between air pollutants and asthma onset remain positive even when attempting to control for misclassification of asthmatics.

A third limitation, inherent to most cohort studies using administrative health databases, is the lack of information on risk factors at the individual level (e.g. socioeconomic status and smoking). We attempted to control for these factors with adjustments of our models using ecological deprivation variables, which are imperfect and may result in residual confounding. Furthermore, our indirect adjustments for exposure to SHS for the Montreal subcohort suggest that point estimates would be marginally influenced by SHS. This is concordant with Villeneuve et al. (2011), who reported an inverse relation between exposures to PM_2.5_ and smoking status in Canada. Other important risk factors for asthma, such as family history of asthma, were not controlled in our analyses.

## Conclusion

Our results support the hypothesis that asthma onset in children is associated with residential exposure to PM_2.5_, O_3_, and NO_2_. Although other risk factors such as SHS may present stronger risks, the pervasive and involuntary nature of exposure to ambient air pollutants may lead to substantial population impacts warranting preventive measures. Future work should address whether residential exposure to air pollutants in the first year of life is also associated with subsequent risk of severe asthma exacerbation.

## Supplemental Material

(485 KB) PDF
