# Supplementary material for: Childhood Exposure to Ambient Air Pollutants and the Onset of Asthma: An Administrative Cohort Study in Québec
Source: Environ Health Perspect. 2016 Jan 5;124(8):1276–82. doi: 10.1289/ehp.1509838 (PMC4977042; doi:10.1289/ehp.1509838)
Supplement: (485 KB) PDF [file ehp.1509838.s001.acco.pdf]

Environ Health Perspect
DOI: 10.1289/ehp.1509838

Note to readers with disabilities: *EHP* strives to ensure that all journal content is accessible to all readers. However, some figures and Supplemental Material published in *EHP* articles may not conform to 508 standards due to the complexity of the information being presented. If you need assistance accessing journal content, please contact ehp508@niehs.nih.gov. Our staff will work with you to assess and meet your accessibility needs within 3 working days.

Supplemental Material

Childhood Exposure to Ambient Air Pollutants and the Onset of Asthma: An Administrative Cohort Study in Québec

Louis-Francois Tétreault, Marieve Doucet, Philippe Gamache, Michel Fournier, Allan Brand, Tom Kosatsky, and Audrey Smargiassi

Table of Contents

Indirect adjustment for second hand smoke

Table S1. Distributions of estimated annual average concentrations of $PM_{2.5}$ and O_3 at both the annual and the birth address in the Montreal sub-cohort

Table S2. Associations between asthma onset and air pollutant levels in Quebec, per increase of an interquartile range in air pollutant levels, without regions of the province where health services may be under-reported

Table S3. Associations between asthma onset and time-varying air pollutant levels, per increase in interquartile range, restricted to non-movers

Table S4. Associations between asthma onset and air pollutant levels, per increase of an interquartile range in air pollutant levels, with reconfirmation of onset when it occurred before the age of five

Table S5. Associations between asthma onset and air pollutant levels, per interquartile range increase in air pollutant levels, stratified by sex

Table S6. Associations between asthma onset and air pollutant levels in Quebec, per increase in interquartile range of pollutant levels, stratified by region

Figure S1. Distribution of the potential bias introduced by second hand smoke on the association between onset of asthma on the island of Montreal and air pollutant levels of: NO_2 (for the years 1996 to 2006) B) $PM_{2.5}$ (for the years 1996 to 2006) and C) O_3 at the birth address (for the years 1999 to 2006)

References

Indirect adjustment for second hand smoke

Individual information on secondhand smoke (SHS) exposure where not available in this study, thus we could not controlled for this potential confounder. However we performed an indirect adjustment for SHS for the Montreal sub-cohort by using a strategy proposed by Steenland and Greenland 2004 and adapted by Villeneuve et al. 2011 for continuous exposures. First, we divided the respective distributions of Montreal NO_2, O_3 and $PM_{2.5}$ exposure estimates into quintiles. For these quintiles of exposure, we estimated the proportion of children exposed at home to SHS. Area-specific (i.e. postal code) prevalence of at home childhood exposure to SHS was retrieved from a survey conducted in 2006 in Montreal (Deger et al. 2010). We also retrieved from a meta-analysis (Tinuoye et al. 2013) a rate ratio representing the association between childhood asthma and SHS. The bias associated with SHS for each quintile was then estimated using the following formula for indirect adjustment (Equation 1):

$$Bias_q = \frac{I_0 \times ((1 - p_{e,q}) + RR_e * p_{e,q})}{I_0 \times ((1 - p_{e,l}) + RR_e * p_{e,l})}$$

where $P_{e,q}$ is the prevalence of exposure to SHS at quintile q, $P_{e,l}$ is the prevalence of exposure to SHS at the lowest quintile and RR_e is the rate ratio of childhood asthma associated with exposure to SHS. I_0 in this equation is the incidence rate of asthma among children unexposed to SHS. However this equation could be simplified to discard the I_0 term. As proposed by Villeneuve et al. 2011, we performed a linear regression in order to present estimation of indirectly adjusted point estimates for a continuous scale. In this model, the estimated bias for each quintile ($Bias_q$) was considered as the dependent variable whereas the independent variable was a random sample of a uniform distribution of air pollutant levels per quintile. The slope of this regression represents an estimation of the potential confounding induced by SHS exposure. Finally, the

indirectly adjusted HRs were calculated by dividing the Montreal sub-cohort HRs, adjusted for sex and the Pampalon deprivation index, by the exponentiation of the bias slope.

In order to compute the uncertainty around our indirectly adjusted point estimates, we used Monte Carlo sampling with 100,000 replications to repeatedly sample from distributions of rate ratios as well as prevalence exposures to SHS. The distribution the rate ratio linking SHS and childhood asthma was based on data from a meta-analysis (n=20) (Tinuoye et al. 2013), we assumed a normal distribution with a mean value equal to the natural logarithm of the rate ratio and standard deviations equal to the standard errors. Identical assumptions were made to construct the distribution around our association between childhood asthma and outdoor air pollutants that were derived from this study. The distribution of the prevalence exposure to SHS was assessed using data from the aforementioned survey (Deger et al. 2010). We presumed a normal distribution with a mean equal to the logit of the proportion of exposed. Adjusted HRs and their 95% Monte Carlo confidence interval were calculated for each pollutant.

Table S1. Distributions of estimated annual average concentrations of $PM_{2.5}$ and O_3 at both the annual and the birth address in the Montreal sub-cohort[a]

	**Birth address**		**Time-varying**	
	**O_3[b]**	**$PM_{2.5}$[c]**	**O_3[b]**	**$PM_{2.5}$[c]**
minimum	12.19	10.10	12.19	9.73
25%	30.11	13.42	29.69	13.00
50%	31.27	13.94	31.27	13.94
75%	32.88	14.40	32.88	14.40
Maximum	38.06	14.85	38.08	14.59
Interquartile range	3.09	0.99	3.18	1.41
Mean	31.55	13.65	31.16	13.62

[a]exposures was based on annual level
[b]for the years 1999-2010 (in ppb)
[c]for the years 1996-2011 (in µg/m^3)
NO_2 levels in the Montreal sub-cohort are presented in table 2 of the article.

Table S2. Associations between asthma onset and air pollutant levels in Quebec, per increase of an interquartile range in air pollutant levels, without regions of the province where health services may be under-reported[a,b,c]

	Sample size	Exposure at birth[c]		Time-varying[d]	
		**Interquartile range**	**Hazard ratios** (95% CI)	**Interquartile range**	**Hazard ratios** (95% CI)
**O_3**[d]	780,593	3.22 ppb	1.11 (1.10, 1.12)[‡]	3.26 ppb	1.13 (1.12, 1.13)[‡]
**$PM_{2.5}$**[e]	1,045,653	6.50 µg/m^3	1.31 (1.28, 1.33)[‡]	6.53 µg/m^3	1.31 (1.29, 1.33)[‡]

[a]associations adjusted for sex and indexes of social and material deprivation
[b]The province of Quebec without the following social and healthcare regions : *North of Quebec, Outaouais, the North Shore, the Gaspésie and the Magdalene*
[c]exposures based on annual levels
[d]for the years 1999 to 2010
[e]for the years 1996 to 2011
[‡]p-value < 0.001

Table S3. Associations between asthma onset and time-varying air pollutant levels, per increase in interquartile range, restricted to non-movers[a,b]

	Sample size	Interquartile range	Hazard ratios (95% CI)
**NO_2**[c]	54,186	5.27 ppb	1.04 (1.01, 1.06)
**O_3**[d]	350,755	3.26 ppb	1.11 (1.10, 1.12)[‡]
**$PM_{2.5}$**[e]	486,815	6.53 µg/m^3	1.33 (1.30, 1.35)[‡]

[a]associations adjusted for sex and indexes of social and material deprivation
[b]exposures based on annual levels
[c]restricted to the Montreal sub-cohort, for the years 1996 to 2006
[d]for the years 1999 to 2010
[e]for the years 1996 to 2011
[‡]p-value < 0.001

Table S4. Associations between asthma onset and air pollutant levels, per increase of an interquartile range in air pollutant levels, with reconfirmation of onset when it occurred before the age of five[a,b]

	Sample size	Exposure at birth		Time-varying exposure	
		**Interquartile range**	**Hazard ratios** (95% CI)	**Interquartile range**	**Hazard ratios** (95% CI)
NO_2[c]	97,536	5.45 ppb	1.06 (1.03, 1.14)‡	5.27 ppb	1.06 (1.02, 1.11)‡
O_3[d]	389,760	3.22 ppb	1.21 (1.19, 1.22)‡	3.26 ppb	1.22 (1.20, 1.23)‡
$PM_{2.5}$[e]	511,187	6.50 $\mu g/m^3$	1.24 (1.22, 1.26)‡	6.53 $\mu g/m^3$	1.23 (1.22, 1.25)‡

[a]associations adjusted for sex and indexes of social and material deprivation
[b]exposures based on annual levels
[c]for the Montreal sub-cohort, for the years 1996 to 2006
[d]for the years 1999 to 2010
[e]for the years 1996 to 2011
‡p-value < 0.001

Table S5. Associations between asthma onset and air pollutant levels, per interquartile range increase in air pollutant levels, stratified by sex[a,b]

	Sex	Sample size	Exposure at birth			Time-varying exposure		
			**Interquartile range**	**Hazard ratios** (95% CI)	**Wald homogeneity test p-value**	**Interquartile range**	**Hazard ratios** (95% CI)	**Wald homogeneity test p-value**
**NO_2**[c]	Female	108,582	5.45 ppb	1.04 (1.02, 1.06)	0.43	5.27 ppb	1.06 (1.03, 1.08)	0.27
	Male	108,164	5.45 ppb	1.05 (1.03, 1.08)‡		5.27 ppb	1.08 (1.06, 1.11)‡	
**O_3**[d]	Female	424,590	3.22 ppb	1.11 (1.09, 1.12) ‡	0.46	3.26 ppb	1.13 (1.11, 1.14)‡	0.41
	Male	404,687	3.22 ppb	1.12 (1.10, 1.13)‡		3.26 ppb	1.13 (1.12, 1.14)‡	
**$PM_{2.5}$**[e]	Female	589,241	6.50 $\mu g/m^3$	1.30 (1.28, 1.31)‡	0.36	6.53 $\mu g/m^3$	1.31 (1.29, 1.33) ‡	0.44
	Male	544,697	6.50 $\mu g/m^3$	1.31 (1.30, 1.33)‡		6.53 $\mu g/m^3$	1.33 (1.30, 1.35)‡	

[a]associations adjusted for sex and indexes of social and material deprivation
[b]exposures based on annual levels
[c]for the Montreal sub-cohort, for the years 1996 to 2006
[d]for the years 1999 to 2010
[e]for the years 1996 to 2011
‡p-value < 0.001

Table S6. Associations between asthma onset and air pollutant levels in Quebec, per increase in interquartile range of pollutant levels, stratified by region[a,b]

	Regions	Sample size	Exposure at birth			Time-varying exposure		
			**Interquartile range**	**Hazard ratios** (95% CI)	**Wald homogeneity test p-value**	**Interquartile range**	**Hazard ratios** (95% CI)	**Wald homogeneity test p-value**
**O_3**[c]	Urban	736,306	3.22 ppb	1.11 (1.10,1.13) ‡.	0.42	3.26 ppb	1.12 (1.09,1.13)‡	0.52
	Rural	92,971	3.22 ppb	1.13 (1.09,1.16)‡		3.26 ppb	1.14 (1.10,1.17)‡	
**$PM_{2.5}$**[d]	Urban	921,062	6.50 $\mu g/m^3$	1.28 (1.24,1.32)‡	0.18	6.53 $\mu g/m^3$	1.30 (1.24,1.33)‡	0.24
	Rural	212,876	6.50 $\mu g/m^3$	1.33 (1.29,1.37)‡		6.53 $\mu g/m^3$	1.36 (1.28,1.48)‡	

[a]associations adjusted for sex and indexes of social and material deprivation
[b]exposures based on annual levels
[c]for the years 1999 to 2010
[d]for the years 1996 to 2011
‡p-value < 0.001

DOI: 10.1289/ehp.1509838

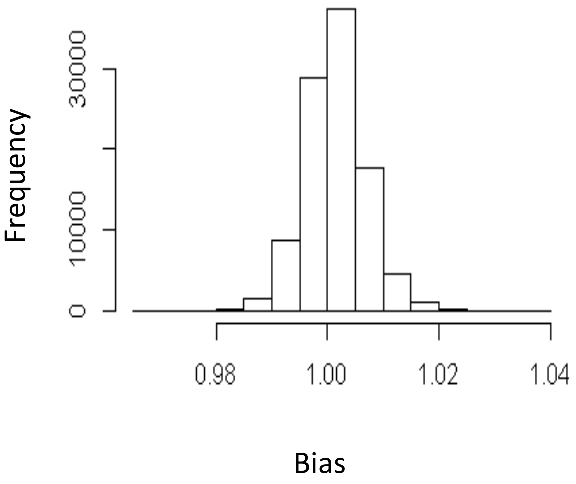

A) Bias factor for NO_2

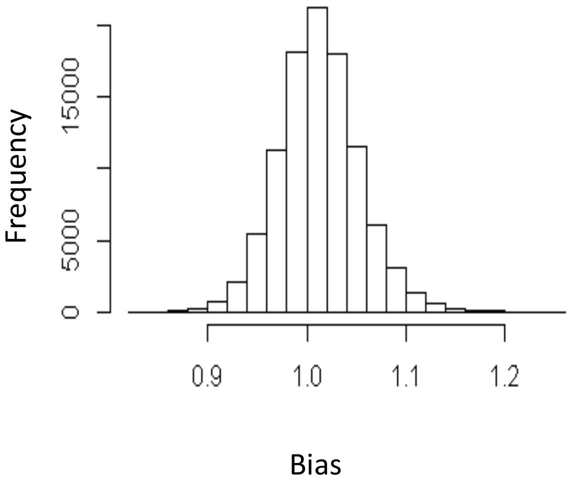

B) Bias factor for $PM_{2.5}$

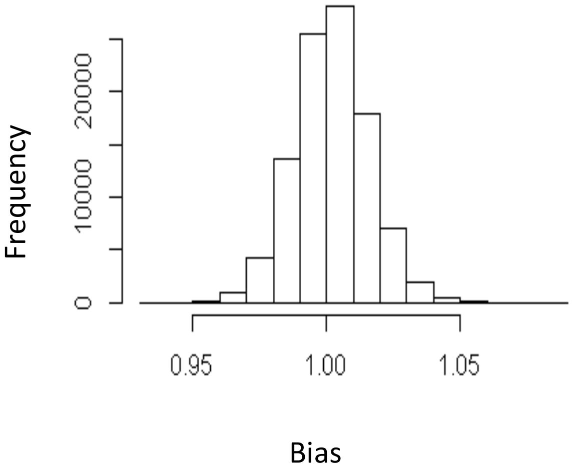

C) Bias factor for O_3

Figure S1. Distribution of the potential bias introduced by second hand smoke on the association between onset of asthma on the island of Montreal and air pollutant levels of: NO_2 (for the years 1996 to 2006) B) $PM_{2.5}$ (for the years 1996 to 2006) and C) O_3 at the birth address (for the years 1999 to 2006)

References

Deger L, Plante C, Goudreau S, Smargiassi A, Perron S, Thivierge RL, et al. 2010. Home environmental factors associated with poor asthma control in Montreal children: A population-based study. J Asthma 47:513-520.

Steenland K, Greenland S. 2004. Monte carlo sensitivity analysis and bayesian analysis of smoking as an unmeasured confounder in a study of silica and lung cancer. Am J Epidemiol 160:384-392.

Tinuoye O, Pell JP, Mackay DF. 2013. Meta-analysis of the association between secondhand smoke exposure and physician-diagnosed childhood asthma. Nicotine & Tobacco Research.

Villeneuve PJ, Goldberg MS, Burnett RT, van Donkelaar A, Chen H, Martin RV. 2011. Associations between cigarette smoking, obesity, sociodemographic characteristics and remote-sensing-derived estimates of ambient pm2.5: Results from a canadian population-based survey. Occup Environ Med 68:920-927.
